# Development and validation of the scale of aesthetics and creativity in chess

**DOI:** 10.3389/fpsyg.2025.1545846

**Published:** 2025-05-30

**Authors:** Alisa Scherbakova, George Engelhard, A. Kadir Bahar

**Affiliations:** ^1^Department of Educational Psychology, Ball State University, Muncie, IN, United States; ^2^Department of Educational Psychology, The University of Georgia, Athens, GA, United States

**Keywords:** aesthetics, creativity, expertise, chess, Rasch analysis, Many-facet Rasch model

## Abstract

This study examined the psychometric quality of the Scale of Aesthetics and Creativity in Chess (SACC) with the data collected from 132 expert and non-expert chess players. To analyze the validity, reliability, and item functioning within the scale, the Many-facet Rasch model was applied. The Rasch measure explained 50.59% of the variance in scores and provided evidence for the scale’s unidimensionality. The reliability indices for items and participants were 0.83 and 0.93, respectively. The expertise level of chess players predicted scores on the SACC; however, Intermediate level players showed the lowest scores in comparison with Expert, Advanced, and Beginner level participants.

## Introduction

The game of chess is a unique domain of expertise that is often considered a combination of sports, science, and art (Chowdhary et al., 2023; Gobet, 2018; Montero and Evans, 2011). Beyond competition and strategy, the game is often appreciated for its aesthetic and creative aspects. Chess competitions traditionally honor games with “brilliancy prizes,” recognizing originality, elegance, and aesthetics. Prominent players often claim that creative expression is one of the major reasons they are attracted to chess (Damsky, 2002; Kasparov, 1987). However, empirical studies of creative thinking in the domain of chess are rather limited (Bilalić et al., 2008; Saariluoma, 1990) because of the lack of recognition of chess as a creative domain and the scarcity of measurement instruments.

This study aims to fill this gap by introducing and validating the Scale of Aesthetics and Creativity in Chess (SACC), a psychometric instrument designed to assess creative and aesthetic qualities in chess problem-solving. To ground this work, we first clarify how creativity and aesthetics are defined, and how these constructs intersect with chess expertise. Also, in addition to reviewing psychological studies of chess creativity and expertise, we analyzed the role of task structure in creative problem-solving, discussed several measurement challenges, and described the development process of the Scale of Aesthetics and Creativity in Chess (SACC).

### Conceptualizing creativity

Creativity is commonly defined through two core criteria: originality and effectiveness (Runco and Jaeger, 2012). Recent models, such as Green et al.’s (2024) process definition, further specify that creativity involves internally focused attention, goal-directed cognitive search, and the generation of solutions not directly retrievable from memory. Neuroscientific research supports this view by highlighting the interplay between the default mode and frontoparietal control networks in facilitating divergent and convergent thinking processes (Beaty et al., 2018; Berry et al., 2017).

Walia (2019) expands on earlier definitions by suggesting that creativity arises from disequilibrium in the environment, prompting individuals to disrupt existing norms to generate novel outcomes. This aligns with the concept of problem sensitivity in early creativity studies (Guilford, 1967; Csikszentmihalyi and Getzels, 1971). Giancola et al. (2022) further emphasize the need to distinguish between process-and product-oriented approaches to creativity, particularly in structured domains like chess.

### Creativity in chess

The question of whether chess requires creativity has been long debated in cognitive psychology and creativity research. While chess is traditionally associated with memory, calculation, and pattern recognition, it also offers ample opportunities for original, surprising, and elegant solutions, especially in complex middle-game positions. Understanding creativity in chess requires examining both the cognitive processes involved in gameplay and the characteristics of the outcomes it produces. From a product-oriented perspective, creativity in chess has been evaluated by considering the originality, effectiveness, and surprise of a given move or sequence (Acar et al., 2017; Cropley and Cropley, 2008; Iqbal, 2012). From a process-oriented perspective, creativity involves divergent thinking to generate multiple possible moves, and convergent thinking to evaluate and refine the most promising ones (Cropley, 2006).

Green et al. (2024) offer a neuroscience-based framework, defining creativity as internally directed cognition constrained by a generative goal. This process engages both the default mode network (associated with spontaneous and associative thinking) and the frontoparietal control network (associated with cognitive control and goal-directed behavior). In chess, these networks are engaged during deep calculation and visualization, especially when players mentally evaluate positions without external aids. Notably, Fuentes-García et al. (2022) found elevated alpha and theta EEG activity in chess experts during blindfolded or rapid chess, patterns often associated with creative cognition (Fink and Benedek, 2014). From a different angle of view, some recent theorists define creativity as an act that arises from the perceived disequilibrium in the environment that requires productive activity and challenging the norms to produce something new (Walia, 2019). During a chess game, players continuously attempt to create disequilibrium for an opponent and use minor weaknesses in their position to gain an advantage. Therefore, sensitivity to problems might play a prominent role in chess.

In one of the earlier studies on creative thinking in chess, Tikhomirov and Vinogradov (1970) utilized think-aloud and eye-tracking methods to explore the process of finding insightful chess solutions. The researchers asked participants to find a correct solution in the end game positions and analyzed verbalized responses. After a short preliminary analysis of the chess positions, participants chose a general strategic plan and verbalized a major hypothesis. The verbalization of the general plan directed the search process and reduced the number of potential solutions. Participants failed to recognize creative solutions as correct if the general plan and hypothesis were wrong. Thus, problem-finding stage of thinking significantly impacted subsequent generation of possible solutions.

Recent studies of creativity in chess focused on exploring the Einstellung effect (Bilalić et al., 2019; Saariluoma, 1990). The Einstellung effect is the phenomenon of sticking to the first idea that comes to mind and resisting shifting the focus to find a more optimal but original solution. Creativity studies revealed that creativity requires recognizing unusual patterns and resisting stereotypy. Bilalić et al. (2008) demonstrated that in a chess position where two solutions are possible, players struggled with finding a less obvious and original one after finding a typical move. Noteworthy, the negative influence of the Einstellung effect on creativity was higher for middle-level players than for experts. Although the reviewed studies suggest that creativity might play an important role in chess, a substantial body of research in the area of expertise revealed that chess skills heavily depend on pattern recognition, memory, and deliberate practice (Ericsson et al., 1993; Gobet, 1997).

### Aesthetics in chess

In the domain of chess, tournaments have a long-lasting tradition of awarding the most beautiful and aesthetically pleasing chess games. The definition of aesthetics remains elusive and largely depends on the domain of study (Ben-Tzur and Feniger-Schaal, 2025; Brooker and Antonini, 2025). In creativity research, aesthetics is defined as the qualities of something that excite admiration and pleasure in an observer (Cropley and Cropley, 2008). Aesthetic appreciation in intellectual domains is usually caused by surprise due to the recognition of an idea as unobvious and effective (Birkhoff, 1933). Empirical studies of chess aesthetics revealed the core components that constitute beauty in chess: originality, surprise, violations of heuristics, and effectiveness in getting the desired advantage (Iqbal, 2012; Margulies, 1977). Therefore, the components of chess aesthetics substantially align with the most widely accepted definitions of a creative product in psychological studies that stress the importance of originality, surprise, and effectiveness (Acar et al., 2017).

Chess theorists and psychologists made numerous attempts to recognize and measure chess aesthetics (Damsky, 2002; Iqbal, 2012; Lasker, 1960; Levitt and Friedgood, 1995; Le Lionnais, 1951; Margulies, 1977; Wilson, 1959) and defined it as what players perceive to be beautiful in the game. Recent empirical studies shifted focus from chess aesthetics to creative problem-solving (Bilalić et al., 2019; Saariluoma, 1990). Although creativity and aesthetics significantly overlap, they have substantial differences that should be further discussed.

Most discussions regarding aesthetics take place in the arts; researchers suggest that elegance, harmony, and symmetry are the major components evoking beholders’ admiration of beauty (Lomas and Xue, 2022; Moore, 1942). However, the phenomenon of aesthetics is not unique to the domains of arts and is applicable across a variety of domains, including science (Perlovsky, 2010; Sinclair, 2001). As German philosopher Immanuel Kant argued, beauty can be found in truth and order (Kant, 1960). Aesthetic appreciation is subjective and depends on the characteristics of the observer and the environment. To evoke surprise and appreciation, an idea should be in the zone of proximal development of the observer: if an idea is too simple or too complex for a beholder, it will not lead to admiration and appreciation. Similarly, a novel product should serve the current needs of humanity to be recognized and appreciated (Cropley and Cropley, 2008). Therefore, aesthetic products might boost one’s sense of growth due to a mind shift.

Chess is one of the domains people derive intellectual pleasure from. The aesthetic value of chess compositions and real games is widely recognized in literature (Iqbal and Yaacob, 2008; Lasker, 1960; Le Lionnais, 1951; Wilson, 1959). World chess champion Emanuel Lasker was one of the first authors who discussed chess aesthetics and suggested that aesthetic chess moves contain elements of surprise, paradox, and effectiveness. According to Lasker (1960), experts and novices can enjoy chess aesthetics since knowledge rather than expertise is required to recognize beauty. However, Lasker (1960) made no distinction in the perception of aesthetics in real chess games and chess compositions.

The first rigorous attempts to measure chess aesthetics were made by judges of chess compositions (Wilson, 1959). Chess composition competitions require chess enthusiasts to create chess positions that can be solved in creative and surprising ways. Although there is usually just one correct solution in such positions, finding it requires original thinking. Wilson (1959) demonstrated that some aspects of aesthetics in chess compositions might be measured; however, his approach was not adapted since human judges were more accurate in their ratings. In the field of psychology, Margulies (1977) was one of the first researchers who studied chess aesthetics empirically. The author asked chess players of different levels to rate the aesthetics of moves in different chess positions. According to Margulies (1977), aesthetic chess moves violated heuristics and won the game economically and the weakest piece. Additionally, experts’ and novices’ ratings were in high agreement. Iqbal (2012) developed a computational aesthetic model to rate chess creativity based on chess aesthetics principles proposed by researchers and chess theorists.

Le Lionnais (1951) analyzed the criteria for which brilliancy prizes for the most beautiful chess games were awarded in chess competitions. The criteria were as follows: Correctness (the combination wins against any defense and is the shortest way to gain an advantage), Difficulty (a degree to which the move challenges an opponent), Vivacity (the move is unexpected and surprising), Originality (the move or sequence of moves is not easily anticipated), Richness (number of original moves and ideas), Logical unity (all moves are subordinated to a major plan). Therefore, chess theorists and researchers agree that aesthetically pleasing chess solutions include the element of surprise, paradox, and effectiveness (Lasker, 1960; Le Lionnais, 1951; Wilson, 1959). Aesthetic intellectual pleasure in chess is a result of surprise after recognizing the originality and effectiveness of the move that was not obvious to the observer. These characteristics of chess aesthetics closely align with the common definitions of creative product (Runco and Jaeger, 2012).

Although aesthetic components in real chess games and chess composition overlap, there are substantive differences that should be taken into consideration. Osborne (1964) suggested that chess is a minor art since the game offers possibilities to create intellectual objects characterized by beauty. Humble (1993) emphasized the role of the competitive aspects of chess in aesthetics. The author asserted that chess is a type of art where opponents co-create beauty in intellectual confrontation rather than collaboration. The presence of an opponent, audience, and time control for choosing a move are the factors that affect aesthetic perception. Since in official chess tournaments, there is time control, players often need to choose a move under time pressure. Consequently, finding an original and unexpected move in a real game can have a more aesthetically profound effect on the opponent and the audience.

Ravilious (1994) argued that chess compositions are a purer form of chess art than real games since chess composers invest a significant amount of time and effort to create a chess composition that has obvious aesthetic value. However, real chess games might be more valuable for studying creative problem-solving. When chess players approach a chess composition, they should think creatively and cannot rely on general principles of good play. However, in such tasks, there is only one correct solution, therefore, the end goal is known. In chess tournaments, opponents strive to find the best move, which is not necessarily an original and unexpected one. Beautiful chess combinations occur rarely and usually are the result of sound play. Therefore, creativity in real chess games is closer to scientific creativity than that in chess compositions.

### Expertise in chess

The remarkable memory of chess experts and their ability to simultaneously play multiple blindfolded games have been widely studied in cognitive psychology (Campitelli and Gobet, 2004). One of the earliest chess studies explored the role of visual imagery in blindfolded games and found that players memorize games as a sequence of moves rather than hold in memory an image of the final position in the game (Binet, 1894). The seminal study conducted by Dutch grandmaster de Groot revealed that chess experts were able to immediately identify the best moves in chess positions due to an enhanced pattern recognition (de Groot, 1946). Later studies investigated differences between novices and experts in recalling chess positions. Experts showed better recall than novices due to memorizing information in larger perceptual chunks. Pieces within a single chunk were connected by mutual defense, proximity, or attacks (Simon and Chase, 1973). As numerous studies have demonstrated, short-term memory can operate 7 ± 2 chunks (Miller, 1956). However, in multiple studies, chess players were able to recall more chunks than predicted by the chunking theory. The chunking theory was further extended to a template theory to explain those differences. The new theory suggested that chess players can recall more chunks due to retrieving complex structures from long-term memory called templates (Gobet and Simon, 1996). As studies of expertise demonstrated, it takes tens of thousands of hours of deliberate practice in any domain to master the necessary skills and build a rich base of templates (Ericsson et al., 1993).

Although pattern recognition, chess knowledge, and hours of practice explain a large proportion of the variance in chess skill levels, the substantial proportion of variance in chess expertise might be accounted for by fluid intelligence, working memory, and personality traits (Blanch and Llaveria, 2021; Smith et al., 2021). The meta-analysis study of deliberate practice across different domains revealed that hours of practice explained only 26% of the variance in chess skill (Macnamara et al., 2014). Another study demonstrated that fluid intelligence explains 16% of the variance in chess memory, thus suggesting that higher-order thinking skills might facilitate conceptual understanding of chess knowledge. The authors assumed that pattern recognition and conceptual understanding both influence chess memory (Lane and Chang, 2018). Other studies in the area of chess and creativity also suggested that fluid intelligence explains a significant proportion of the variance in chess skills as well as creative abilities (Burgoyne et al., 2019; Gerwig et al., 2021).

Personality traits have been found to be an important predictor of chess skill in expert and novice populations. For instance, novice chess players with higher scores on openness to experience and extroversion were more likely to be attracted to chess (Bilalic et al., 2007). Expert players scored higher than the general population on scales of unconventional thinking, sensation seeking, and expressive suppression, and lower on measures of neuroticism (Joireman et al., 2002). Numerous studies suggested that openness to experience and sensation seeking are strong predictors of creativity (Twomey et al., 1998; Xu et al., 2021).

A new avenue of research explored chess personalities defined as preferences for attack or defense strategies during different stages of the game. Computer simulations demonstrated that players tend to make more mistakes when playing against an opponent with the opposite chess personality profile. Players of an attacking style prefer complex positions with many pieces on the board under mutual threats. Meanwhile, players of defense style prefer to reduce the number of pieces on the board by exchanging them and rely more on strategic planning than on tactics (Dhou, 2018). Therefore, the personality profiles of chess players might reflect their preferences for different task structures.

The choice of the best moves in chess is a generative goal since the number of possible games is infinite, and players make choices depending on chess personality profiles and opponents’ skill level (Dhou, 2019; Holdaway and Vul, 2021). Although during the game players’ attention is focused on external stimuli, they are not allowed to move the chess pieces on the board and instead should analyze the position and calculate the possible lines in their mind. Therefore, the thinking process in chess might not fully satisfy the first criterion of internally focused attention in the process of creative thinking as described by Green et al. (2024). However, the studies of neuropsychological differences in playing different chess modalities showed that expert-level players exhibit greater electroencephalogram alpha and theta wave power during blindfolded games, which are associated with the creative thinking process (Fink and Benedek, 2014).

### The role of task structure in creative problem-solving

Most of the early chess studies were exploratory and did not recruit large samples of chess players. Instead, researchers focused on in-depth analysis of problem-solving processes (Binet, 1894; de Groot, 1946; Chase and Simon, 1973). One of the significant but not addressed limitations of chess studies is the lack of discussion regarding the influence of the task structure on the thinking process. The structure of the task might significantly impact the creative problem-solving performance (Bahar, 2013; Bahar and Maker, 2015). Task structure in chess can be defined based on several parameters: (1) cognitive load, which depends on the number of chunks operated in a working memory and the connections between them (Sweller et al., 2011); (2) required cognitive processes (recall, problem-solving); (3) depth of needed calculations; (4) the goals: well-defined (checkmate in two moves) or ill-defined (finding the best move during the game).

For instance, de Groot (1978) in his study used chess positions from tournament games with complex interactions between pieces. de Groot (1978) concluded that grandmasters and weaker masters did not significantly differ in the depth of search, and pattern recognition played a more prominent role in performance. These conclusions were not critically examined for many years. Campitelli and Gobet (2005) assumed that the chess positions in de Groot’s study (1978) did not require a sufficient depth of search, and that is the reason why the study did not find the skill effect on depth of search. Campitelli and Gobet (2005) administered three complex chess positions that required considerable search and evaluation to three players of different skill levels. The results revealed a significant skill effect on time and depth of search.

Tikhomirov and Vinogradov (1970) explored the creative thinking process of chess players using endgame chess positions with a well-defined end goal. In such positions, cognitive load might be lower due to fewer number of pieces. Participants were instructed to find the only correct solution, which can be achieved through restructuring and subsequent insight. Thus, positions required a shift of perspective rather than depth of search or pattern recognition. Moreover, such positions in real games are rare, and even when they happen, the end goal is not as well-defined as in the case of solving chess puzzles.

Chess can be used as a tool to study creative thinking processes in a well-controlled laboratory environment using task structures that are most likely to elicit original thinking. Additionally, due to the sophisticated measurement scale of the chess skill level (Elo, 1978), researchers can examine the relationship between expertise and creativity. However, assessing creativity in chess remains challenging, and only a scarce number of empirical studies have attempted to develop reliable measurement instruments. In the next section, we will discuss attempts to measure chess creativity by researchers and chess players.

### Need for a domain-specific scale

Domain-specific research suggests that creativity is heavily influenced by context, prior knowledge, and the customs of a given field (Baer, 2012; Bahar et al., 2024). What qualifies as original or effective can vary significantly across disciplines, which underscores the importance of both cognitive mechanisms and domain expertise. From a domain-specific standpoint, the study of creativity in chess is relatively sparse. Moreover, while domain-general creativity measures exist, they often fail to predict real-world creative performance in specific fields (Baer, 2012; Barbot et al., 2019). Given the complex interplay of strategy, aesthetics, and expertise in chess, a domain-specific scale is needed. The SACC aims to address this by evaluating six principles: originality, violation of heuristics, sacrifice of material, economical win, correctness, and employment of chess themes. These principles are grounded in both creativity and chess research, as discussed above. In the following sections, we detail the development and validation of the SACC, describe our methodological procedures, and examine its psychometric properties using the Many-Facet Rasch Model.

## Scale development: measuring aesthetics and creativity of chess solutions

While developing the SACC, we considered the measurement issues for the assessment of creativity in chess reviewed in the previous sections. Thus, considering the challenges, we focused on the following aspects: selection of the tasks and providing instructions that are close to real-game situations. Additionally, we developed instructions for expert raters to assess the creativity of the solutions.

### Selection of the task and task instructions

**Figure 1:**
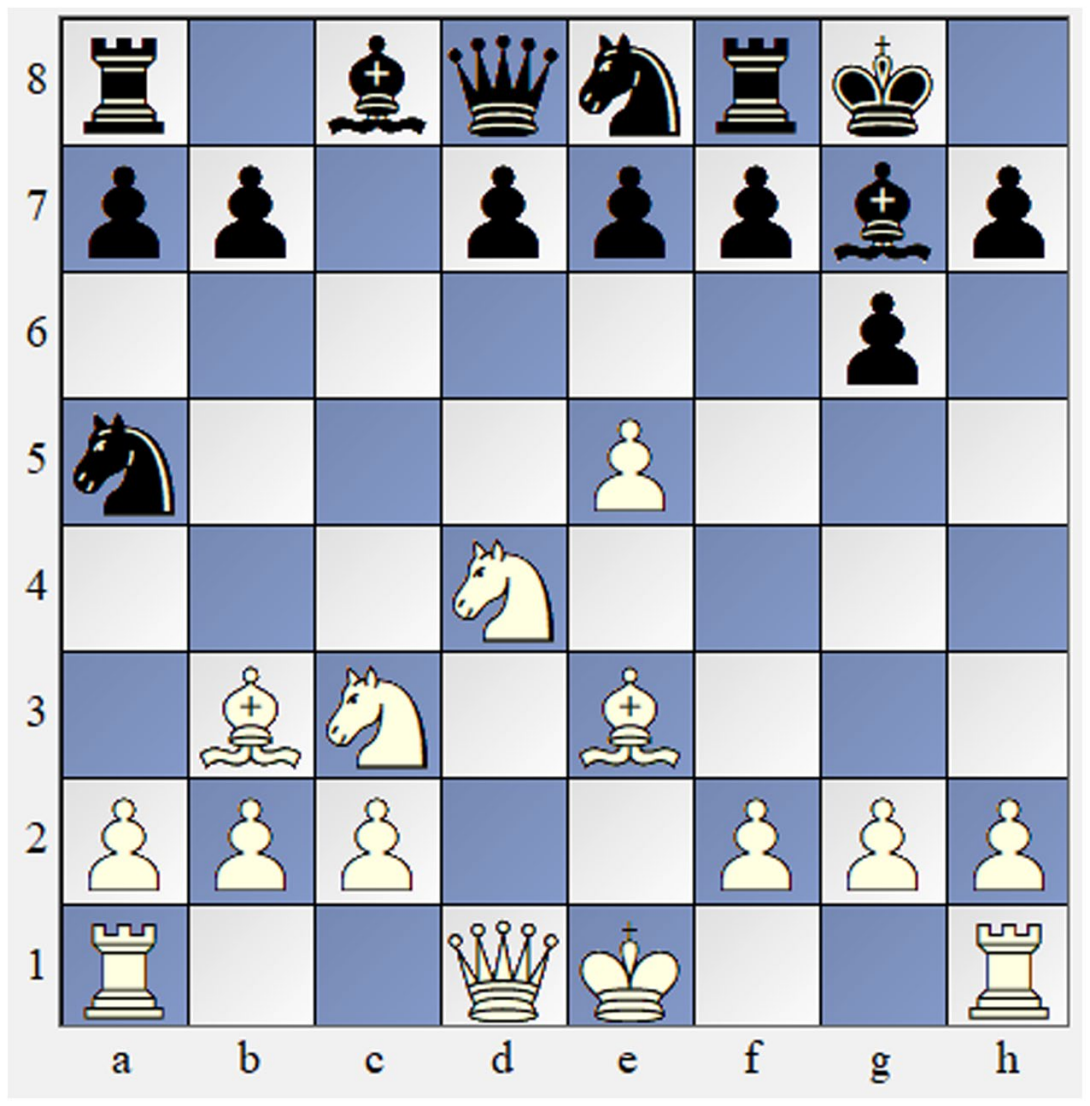
Chess problem 1. Solution: 1. B:f7-Kf7 2. Ne6—Ke6 3. Qd5—Kf5 4. g4 +/−.

As reviewed chess studies have demonstrated, the structure of the task and instructions define the direction of the thinking process. For the goals of the present study, we selected opening and middle-game chess positions that are often encountered in real games. All of them seemed theoretical and did not have an obvious solution. For instance, in the first task, participants were asked to choose the strongest move in the position borrowed from the famous Fisher vs. Reshevsky game in 1958 (Chessgame.com, n.d.; See Figure 1). This position seems theoretical at first, but it has an unobvious paradoxical and the strongest move that gains an advantage. To make tasks semi-open, we asked participants to analyze positions and choose a move they would have played in a real-life game. Participants were not informed that each position had a single original and effective solution.

### Development of the scoring process

To construct a measure of the creativity of the chess solutions, we outlined six dimensions and mapped them on related creativity, aesthetic, and chess constructs to provide evidence of the construct validity of the instrument (Table 1). Chess composition conventions and brilliancy characteristics of the real games have common general aesthetic principles; for the goals of the present study, we included the following six dimensions:

- violation of heuristics,
- economical win,
- sacrifice of material,
- employing chess themes,
- correctness, and
- originality

**Table 1:** Rubric for chess creativity dimensions and construct mapping.

Chess aesthetic principle	Descriptive definition	Construct mapping
Violates heuristics successfully	The move sequence violates common principles of good play, such as: keeping your king safe, not leaving your pieces under attack, capturing an opponent’s material, increasing the mobility of the pieces.	**Chess construct**: Margulies (1977) most moves that violate heuristics are ineffective, but those that are both unusual and effective are considered beautiful. **Creativity construct**: Amabile (1983), surprise as a result of a not non-algorithmic thinking.
Wins economically	The move sequence is the shortest leading to an advantage and does not use more pieces than necessary to achieve the goal.	**Chess construct**: Iqbal and Yaacob (2008) using not more material than needed to achieve the desired outcomes. **Aesthetics construct**: Cropley and Cropley (2008) internal elegance of the well worked ideas.
Sacrifices material	Exchanging a stronger piece for a weaker one to gain an advantage.	**Chess Construct**: Damsky (2002) Sacrifice refers to an unequal exchange of pieces that later leads to advantage.
Correctness	The proposed solution works against any defense.	**Chess Construct**: Lasker (1960), the move or move sequence unavoidably leads to an advantage. **Aesthetics Construct**: Cropley and Cropley (2011), the product or idea is logical and useful.
Originality	The solution is not typical for positions of such type.	**Chess Construct**: Damsky (2002), Rareness of the move or move sequence in similar chess positions. **Creativity Construct**: Osborn (1953) Rarity of the idea in the specific context.
Employs chess themes	The solution employs tactical themes such as fork, pin, skewer, X-ray, etc.	**Chess Construct**: Margulies (1977) themes constructed with chess elements.

Scores: 1—low, 2—somewhat low, 3—average, 4—somewhat high, 5—high.

Violation of heuristics requires disregarding common chess principles, such as not leaving pieces under attack, controlling the center, developing the pieces, and protecting the king (Margulies, 1977). This chess principle relates to the construct of surprise in creativity literature, which was defined as a result of non-algorithmic thinking that relies on heuristics. Economical win principle assumes that the advantage in the game was achieved with less material or by using the weakest pieces (Iqbal and Yaacob, 2008). This chess principle is similar to the aesthetic construct of internal elegance of well-worked ideas (Cropley and Cropley, 2008).

We also included purely chess principles such as Sacrifice and Employing Chess. Sacrifice of the material is an exchange of pieces of unequal value, which leads to gaining a larger advantage after the sequence of paradoxical moves (Damsky, 2002). Employing chess themes principles includes using chess tactics, which are short sequences of moves that lead to gaining an advantage (Margulies, 1977). The correctness principle in chess requires the sequence of moves to work against any defense, and is similar to the creativity construct of usefulness (Cropley and Cropley, 2011). Finally, originality is the sequence of moves that have never been seen before and can be considered rare (Damsky, 2002). Statistical rarity of the idea is a related creativity construct (Osborn, 1953). However, originality should not be equated with violation of heuristics since some of the heuristics are common and widely known. Similarly, although correctness and effectiveness are similar constructs, original and effective solutions can be proven wrong if the defense is possible.

Two chess Grandmasters and a Candidate Master (FIDE Elo ratings 2,509, 2,459, and 2,004, respectively) were recruited to rate chess solutions according to the rubric of the scale (Table 1). Creativity researchers suggest that raters should have a sufficient level of expertise in the domain (Amabile, 1982; Baer and McKool, 2014). All raters had an official chess rating, which is an accepted measure of expertise based on performance in tournaments. Additionally, the raters have at least five years of coaching experience with students of different levels. Coaching experience allows raters to judge performance depending on participants’ level of expertise.

**Figure 2:**
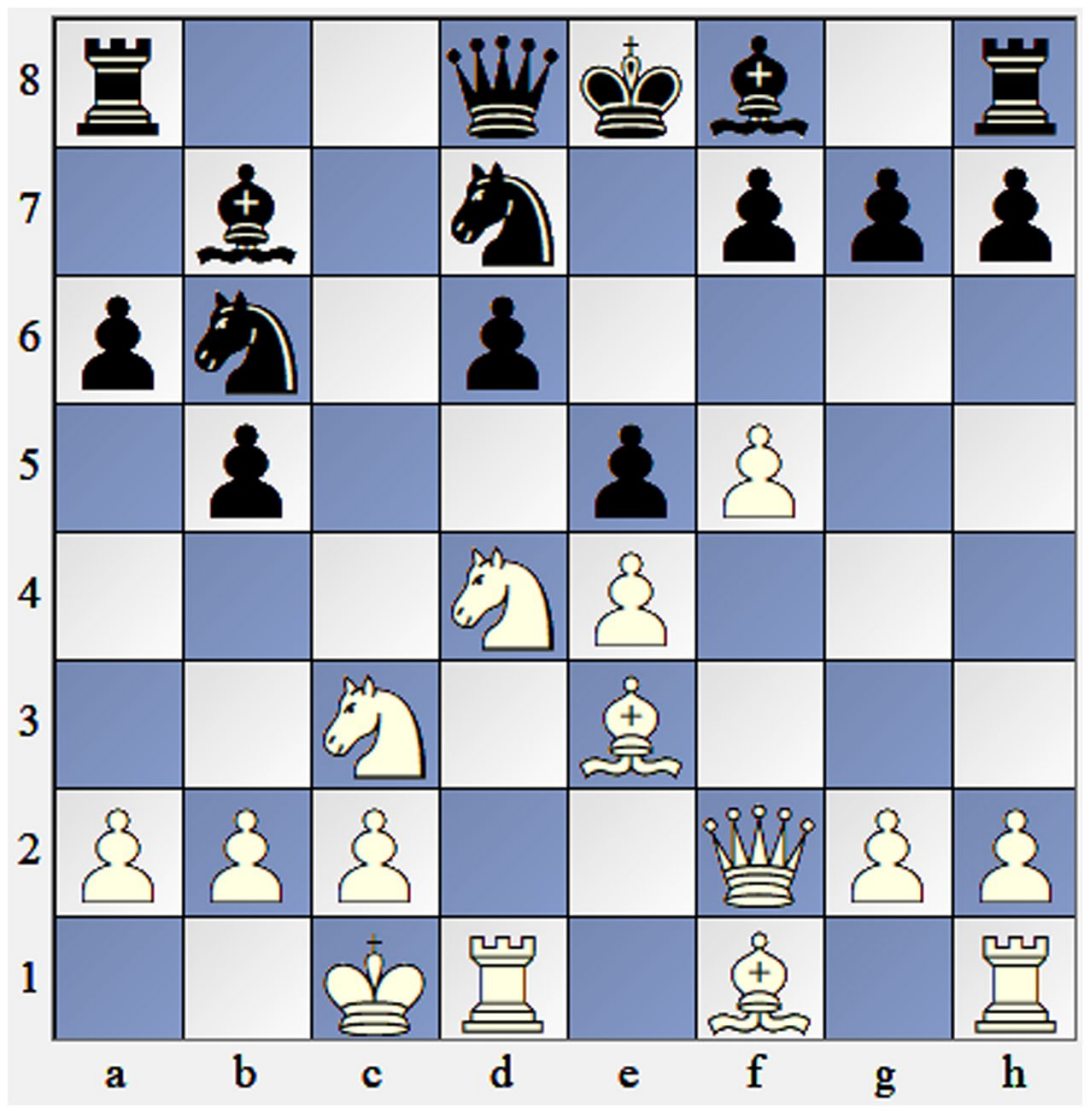
Chess problem 2. Solution: 1. Ne6—fe 2. fe—kf6 3. Bb6 + −.

**Figure 3:**
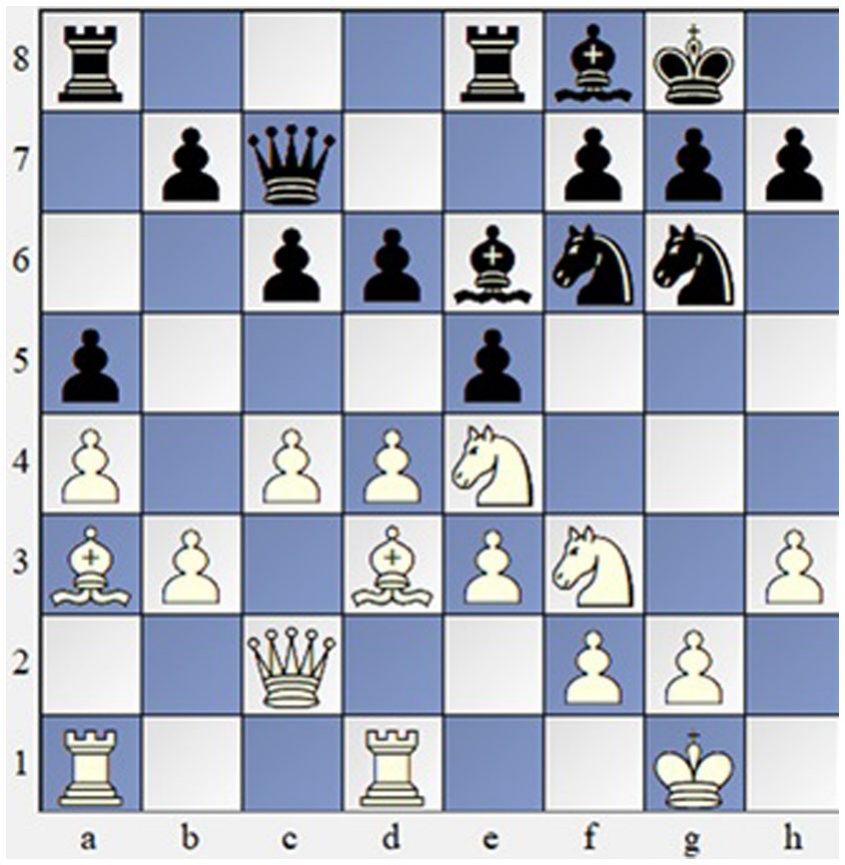
Chess problem 3. Solution: 1. N:d6 (or B:d6)—B:d6 2. B:d6—Q:d6 3. De—N:e5 4. B:h7 + +−.

For the goals of the present study, we used three chess positions and asked participants to choose the best sequence of moves (Figures 1–3). Given the large sample size and the overall number of generated responses, we collected all unique solutions for every chess problem. Every position had approximately 9–18 unique solutions. Then, three raters were asked to assess the solutions for every move sequence on a scale of 1–5 based on the rubric (Table 1). Raters worked independently and without guidance and were asked to assess components considering the sample and context. Before rating responses, judges were instructed to analyze chess positions and choose the best move, then they were given the correct creative solutions. Raters provided highly reliable ratings (*α* = 0.936).

### Research questions

The goal of the study was to examine the psychometric quality of the Scale of Aesthetics and Creativity in Chess (SACC) using the Many-facet Rash model analysis. To analyze the external validity of the scale, chess categories were included as a measure of skill level. The present study answers the following research questions:

1. To what extent, the SACC validly and reliably measure chess creativity and aesthetics?
2. Does the SACC function in the same way across different levels of chess expertise?

## Methods

### Participants

The present study collected responses from *N* = 132 adult chess players living abroad and in the USA. The recruit participants we sent invitations via email to the chess clubs across the USA and posted an invitation on the Reddit platform. The contact information of chess clubs was retrieved from the US Chess Federation online database. The chess club hosts received a flyer with a detailed description of the study and a link to the Qualtrics survey.

The mean age of the participants was 29.86 years (*SD* = 13.60), and the mean number of years playing chess was 14.05 (*SD* = 15.59). The gender demographics of the sample were as follows: Males (*N* = 122), Females (*N* = 5), Other (*N* = 4). Players reported their official regular chess ratings on the International Chess Federation (FIDE) or United States Chess Federation (USCF) Elo interval scale. The Elo rating system (Elo, 1978) measures the skill level of chess players based on their performance in rated chess competitions. The mean of the Elo rating scale is 1,500 (*SD* = 200). Players with a rating higher than 2,000 Elo are usually called Experts, while Beginner level chess players are usually unrated or have ratings in the range from 800 to 1,100 points.

To ensure sufficient precision in parameter estimation, we conducted a power analysis using the *raschpower* package in R (version 4.3.3) to evaluate whether a sample size of 132 participants would provide adequate power for detecting item misfit in a Rasch model developed for our instrument assessment. The simulation included 1,000 replications, with item discrimination set to 1.0 and a moderate item misfit effect size of 0.5. The analysis indicated that the design achieved an estimated power of approximately 0.82 at an alpha level of 0.05, suggesting sufficient sensitivity to detect misfitting items. These results support the adequacy of the planned sample size for the purposes of test development and ensure acceptable precision in the estimation of item parameters (Linacre, 1994; Wright and Masters, 1982).

Participants fell into 6 chess rating categories: (1) Unrated (*N* = 63), (2) Beginner (*N* = 8), (3) Intermediate (*N* = 10), (4) Intermediate II (*N* = 10), (5) Advanced (*N* = 24), (6) Expert (*N* = 17). The mean rating of rated players was (*M* = 1727.39, *SD* = 413.32). The chess rating categories were assigned according to the official chess ratings (Table 2). Amateur unrated chess players comprise the vast majority of the chess players’ population worldwide. However, unrated players’ skill level might widely vary depending on the years of practice. According to the United States Chess Federation, among rated players, Experts are placed in the 96th percentile, while players with an Elo rating below 1,100 are placed in the 52nd percentile. Additionally, the author (candidate master) and two grandmaster-level chess players rated the creativity of the solutions based on the provided rubric (Table 1).

**Table 2:** Chess rating categories.

Characteristic	Chess rating levels (Elo, 1978)	Result, *N* (%)
Chess rating categories		
Unrated	Unrated	63 (47.7)
Beginners	<1,100	8 (6.06)
Intermediate I	1,101–1,400	10 (7.57)
Intermediate II	1,401–1,700	10 (7.57)
Advanced	1,701–2,000	24 (18.28)
Experts	2,000>	17 (12.87)
Total		132 (100.0)

### Instrument

#### The scale of aesthetics and creativity in chess (SACC)

Participants were asked to choose the best move in three chess positions of different levels of difficulty (Figures 1–3). The overall time limit for each chess position was 5 min. Responses of the participants were then independently rated by three chess experts across 6 dimensions: violation of heuristics, economical win, sacrifice of material, employing chess themes, correctness, and originality (Table 1). Raters assigned values from 1 to 5 across six dimensions to all solutions and reached a high level of inter-rater reliability with *α* = 0.936.

### Procedures

We asked the hosts of several chess clubs to share a call for volunteer participants that included a link to a Qualtrics page with a survey. Participants were asked to sign a consent form and to read information regarding the risks, benefits, and time commitment. After agreeing to take part in the study, participants reported demographic information regarding age, years that they played chess, gender, and official chess ratings.

After providing demographic information, participants were presented with the first problem of the SACC. Chess players had a five-minute time limit per problem. The directions asked raters to preview chess positions to subjectively judge the difficulty of each problem. Independent expert raters were instructed and trained to rate solutions to each problem across 6 dimensions on the scale from 1 to 5 (1—low, 2—somewhat low, 3—average, 4—somewhat high, 5—high) using the rubric in Table 1.

### Data analyses

To examine psychometric qualities of the SACC, we used invariant measurement based on Rasch Measurement Theory (Engelhard, 2013; Engelhard and Wang, 2021). The Rasch model enables researchers to simultaneously evaluate both items and participants to determine the item difficulty on a variable map that illustrates the position of items and persons on the scale and identifies which items were the hardest to endorse by participants (Rasch, 1960/1980; Wright and Masters, 1982).

To analyze the dimensionality of the scale and functioning of the items, we utilized the Facets program (Linacre, 2017). Rasch (1961) emphasized that measurement should focus on individuals rather than being centered on groups. The primary goal of the Rasch model is to derive item-difficulty estimates and analyze the impact of additional explanatory variables. Therefore, the Rasch model (1960/1980) includes two parameters: the ability of person *n* (*θ*_n_), and item difficulty (δ_i_). If a person n responds to item i, the probability of that person being correct on that item is:


$$P_{\mathrm{ni}1}=\frac{\exp(\theta_{n}-\delta_{i})}{1+\exp(\theta_{n}-\delta_{i})},$$

where P n_i_1 is the probability of person n scoring 1 on item i.

Rasch analysis estimates the degree to which data conforms to the model instead of seeking a model that fits the data. In cases where data does not fit the model, individual responses and items are examined for inconsistencies. Obtained scores can be located on a logit scale and compared between individuals and items. Rasch Many-facet model can be utilized for polytomous items with the shared structure across all items (Andrich, 1988). For the goals of the present study, we included the chess rating categories as an explanatory variable in our analysis:


$$\ln\frac{P_{\text{nijmk}}}{P_{\text{nijmk}}-1}=\theta_{n}-\delta_{i}-\alpha_{j}-\gamma_{m}-\tau_{k}$$

where P*nijmk* is the probability of player *n* responding in category *k* on item *i*; Pnijmk−1 is the probability of player *n* responding in category *k* − 1 on item *i*; *θ* is the level of chess creativity for player *n*; δ_i_ is the difficulty of item i, *α*_j_ represents chess rating category; *γ* represents the task, and τ_k_ indicates the probability of being observed in chess rating category *k* relative to category *k* − 1.

## Results

### Item descriptive statistics and inter-item correlations

First, we explored descriptive statistics, including means and standard deviation scores across all dimensions of the scale for three chess problems (Tables 3–5). Problem 2 was the easiest for participants, with mean scores ranging from (*M* = 3.5, *SD* = 0.44) to (*M* = 5.0, *SD* = 0.00) on a 5-point scale. Problem 3 was the most challenging, with scores ranging from (*M* = 2.7, *SD* = 0.34) for Intermediate II players and (*M* = 4.4, *SD* = 0.19) for Experts. Intermediate I players showed the lowest average scores across all three chess problems, while the opposite was true for Expert players.

**Table 3:** Descriptive statistics problem 1.

Chess category	Means (*SD*)						
	Heuristics violation	Economic win	Sacrifice	Correctness	Originality	Themes	Average
Unrated	4.0 (0.18)	3.4 (0.17)	3.4 (0.20)	3.1 (0.20)	3.5 (0.20)	3.2 (0.21)	3.4 (0.17)
Beginners	4.2 (0.41)	3.0 (0.50)	3.25 (0.49)	2.4 (0.60)	3.4 (0.56)	2.6 (0.65)	3.1 (0.46)
Intermediate 1	3.1 (0.52)	3.1 (0.43)	2.6 (0.58)	3.4 (0.37)	2.7 (0.56)	2.6 (0.59)	2.9 (0.49)
Intermediate 2	3.6 (0.58)	3.3 (50)	3.3 (0.56)	3.2 (0.53)	3.3 (0.56)	3.3 (0.56)	3.3 (0.51)
Advanced	3.9 (0.34)	3.8 (0.30)	3.7 (0.36)	3.9 (0.29)	3.7 (0.37)	3.7 (0.37)	3.8 (0.32)
Experts	4.9 (0.05)	4.9 (0.11)	4.9 (0.19)	4.8 (0.17)	4.9 (0.12)	4.8 (0.11)	4.9 (0.11)

**Table 4:** Descriptive statistics problem 2.

Chess category	Means (*SD*)						
	Heuristics violation	Economic win	Sacrifice	Correctness	Originality	Themes	Average
Unrated	4.4 (0.11)	3.7 (0.20)	4.2 (0.15)	3.65 (0.21)	4.0 (0.16)	3.5 (0.20)	3.9 (0.15)
Beginners	4.75 (0.16)	3.7 (0.62)	4.7 (0.16)	3.75 (0.62)	4.25 (0.37)	3.9 (0.55)	4.2 (0.40)
Intermediate 1	4.5 (0.16)	3.1 (0.52)	4.4 (0.22)	3.00 (0.56)	3.8 (0.33)	3.2 (0.50)	3.7 (0.36)
Intermediate 2	4.1 (0.38)	3.1 (0.53)	4.1 (0.38)	3.10 (0.53)	3.6 (0.43)	3.10 (0.53)	3.5 (0.44)
Advanced	4.7 (0.17)	4.1 (0.32)	4.7 (0.17)	4.13 (0.32)	4.4 (0.22)	4.1 (0.30)	4.4 (0.23)
Experts	5.0 (0.00)	5.0 (0.00)	5.0 (0.00)	5.0 (0.00)	5.0 (0.00)	5.0 (0.00)	5.0 (0.00)

**Table 5:** Descriptive statistics problem 3.

Chess category	Means (*SD*)						
	Heuristics violation	Economic win	Sacrifice	Correctness	Originality	Themes	Average
Unrated	2.06 (0.15)	4.0 (0.09)	2.35 (0.21)	4.34 (0.07)	2.3 (0.15)	3.0 (0.17)	3.0 (0.13)
Beginners	2.3 (0.49)	4.4 (0.18)	2.7 (0.67)	4.6 (0.12)	2.4 (0.54)	3.5 (0.43)	3.3 (0.40)
Intermediate 1	2.0 (0.43)	3.6 (0.34)	2.1 (0.50)	3.9 (0.36)	2.4 (0.40)	2.5 (0.48)	2.8 (0.33)
Intermediate 2	1.8 (0.37)	3.9 (0.23)	2.0 (0.50)	4.2 (0.19)	2.0 (0.40)	2.6 (0.46)	2.7 (0.34)
Advanced	2.7 (0.27)	4.3 (0.16)	3.1 (0.38)	4.5 (0.13)	2.8 (0.31)	3.7 (0.29)	3.5 (0.25)
Experts	3.6 (0.22)	4.8 (0.09)	4.4 (0.31)	4.9 (0.05)	3.9 (0.26)	4.6 (0.21)	4.4 (0.19)

Then, we explored inter-item correlations across six dimensions of the scale and chess rating categories (Table 6). Chess rating category had the lowest correlation with the Heuristics Violation dimension (*r* = 0.10), and the highest with Correctness (*r* = 0.34). We conducted Fisher’s z-test to compare correlation coefficients between chess rating categories and scores on Heuristics Violation and Correctness dimensions of the scale. The analysis revealed that the difference was statistically significant with *z* = 2.25, *p* = 02. The Heuristics Violation dimension had an extremely high correlation with Originality and Sacrifice items (*r* = 0.93 for both). The Economic Win and Correctness dimensions also showed a high level of inter-item correlation (*r* = 0.94).

**Table 6:** Bivariate correlations among scale dimensions and chess rating categories.

Dimensions	Mean (*SD*)	Chess rating category	Heuristics violation	Economic win	Sacrifice	Correctness	Originality	Themes
Chess rating category	2.8 (2.0)	1.00						
Heuristics violation	3.6 (1.5)	0.10	1.00					
Economic win	3.9 (1.3)	0.28**	0.43**	1.00				
Sacrifice	3.6 (1.6)	0.23**	0.93**	0.58**	1.00			
Correctness	3.9 (1.4)	0.34**	0.22**	0.94**	0.42**	1.00		
Originality	3.4 (1.5)	0.20*	0.93**	0.65**	0.95**	0.47**	1.00	
Themes	3.5 (1.6)	0.26**	0.73**	0.86**	0.85**	0.74**	0.86**	1.00

**p < 0.01.

### Inter-rater reliability across dimensions

To explore inter-rater reliability among three raters, we computed Cronbach’s alphas (Cronbach, 1951) for each dimension of the scale (Table 7). Raters have demonstrated high levels of agreement across the following dimensions: Sacrifice (*α* = 0.874), Correctness (*α* = 0.897), and Employment of Chess Themes (*α* = 0.846). The lowest level of agreement was observed for the Originality dimension with (*α* = 0.627).

**Table 7:** Reliability coefficient across scale dimensions.

Scale dimension	Cronbach’s *α*
Heuristics violation	0.741
Economical win	0.703
Sacrifice	0.874
Correctness	0.897
Originality	0.627
Themes	0.846

### Many-facet Rasch model analysis of the SACC

The many-facet Rasch model included four facets: participants, items; and two explanatory variables: category of chess ratings, and the task. According to the Rasch model (Rasch, 1960/1980), the difficulty of the item depends on how hard it is for participants to answer the item correctly. The variable map shows logit scale values for the items; large positive values show that the item was hard to answer for subjects and reflect the high levels of creativity in chess. The Rasch model explained 50.59% of the variance in the data and demonstrated unidimensionality of the scale. Indexes of reliability for items and participants were 0.83 and 0.93, respectively (Table 8).

**Table 8:** Rasch summary statistics for persons, items and chess rating categories.

Statistics	Persons	Items	Chess rating category
Measure			
*M*	3.64	3.64	3.63
SD	1.01	0.21	0.60
*N*	132	6	6
Outfit			
*M*	0.94	0.94	0.92
SD	0.56	0.29	0.23
Infit			
*M*	0.95	1.04	1.01
SD	0.36	0.27	0.18
Separation statistic			
Reliability of separation	0.83	0.93	0.98
*χ^2^*	774.0	71.9	194.0
*Df*	131	5	5
Variance explained by Rasch measures	50.59%		

**Figure 4:**
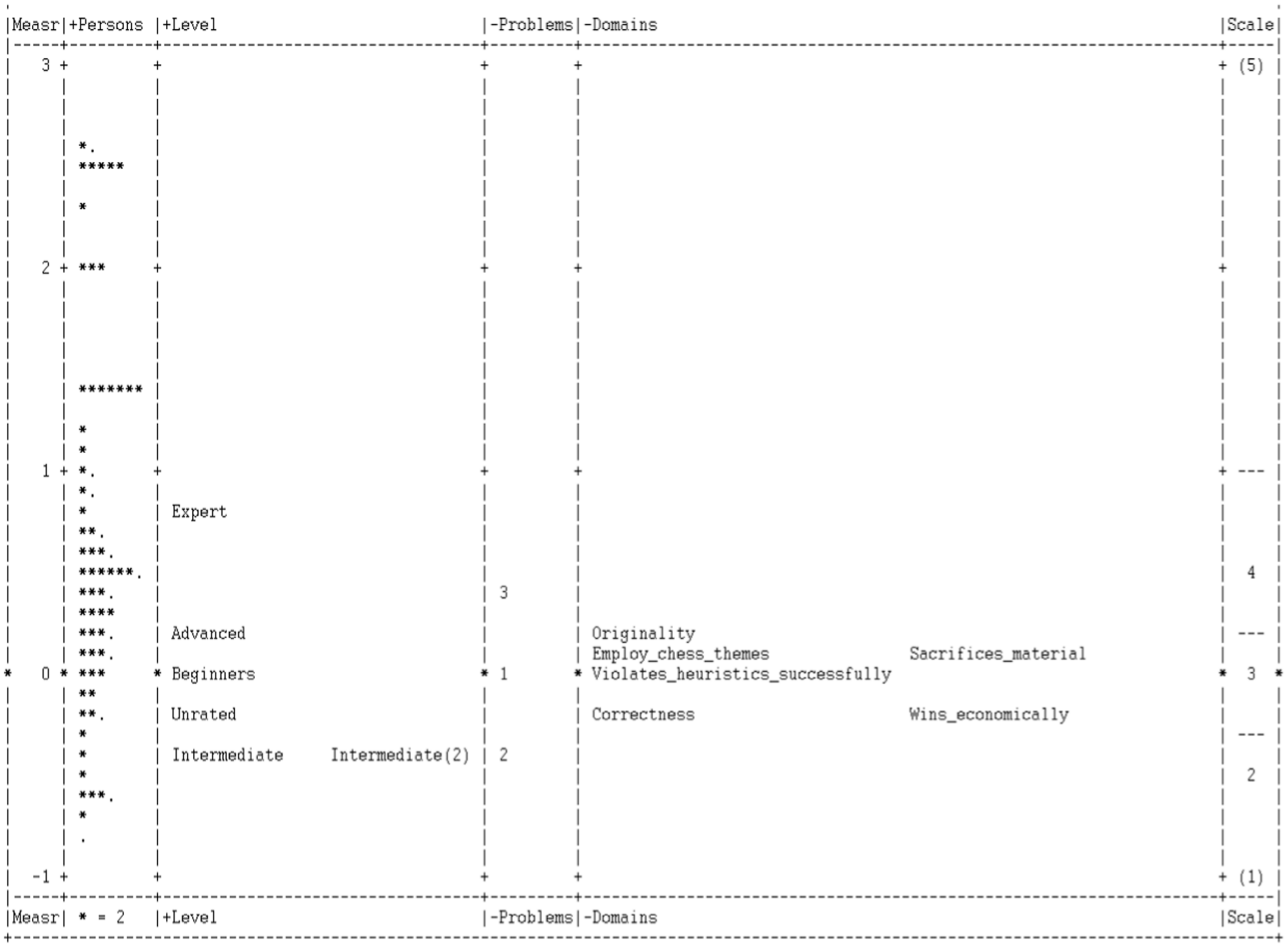
Wright Map.

The Facets program produced the Wright Map, which visually represents items and subjects on the scale. The Wright Map was used to examine the overall functioning of the scale and the difficulty of the specific items. The Wright Map showed a good spread of locations across participants and items. The Wright Map (Figure 4) revealed that the Originality item was the hardest to endorse, while Correctness and Economic Win items were the easiest for participants. Expert-level chess players received the highest scores, while intermediate-level players received the lowest scores, even in comparison with the categories of Beginners and Unrated chess players.

To examine the functioning of the items, we analyzed Infit and Outfit statistics, which demonstrate how well the data fits the Rasch Model. According to Engelhard and Wind (2018), the values higher than 0.50 and lower than 1.50 can be interpreted as productive for the measurement. Values below 0.50 are less productive but do not distort the measure. The values higher than 1.50 but lower than 2.00 are unproductive for the measurement but do not distort the scale. And items with values over 2.00 are unproductive for the measurement and distort the measure (Table 9). Overall, all items demonstrated high fit to the Rasch Model with Outfit and Infit statistics in the range from 0.78 to 1.55 and from 0.76 to 1.45, respectively (Table 10). The item Correctness showed slight overfit to the model, with the Infit value equal to 1.55.

**Table 9:** Guidelines for interpreting mean squared error (MSE).

Mean squared error (MSE)	Interpretation	Fit category
0.50 ≤ MSE < 1.50	Productive for measurement	A
MSE < 0.50	Less productive for measurement, but not distorting of measures	B
1.50 ≤ MSE < 2.00	Unproductive for measurement, but not distorting of measures	C
MSE ≥ 2.00	Unproductive for measurement, distorting of measures	D

**Table 10:** Mean squared error fit category.

Items	Measure	S.E.	Infit MSE	Fit category	Outfit MSE	Fit category	Pt Bis	Disc
Heuristics	0.04	0.05	0.97	A	0.93	A	0.40	1.06
Economy	−0.22	0.05	1.13	A	1.06	A	0.39	0.69
Sacrifice	0.07	0.05	0.89	A	0.63	A	0.45	1.50
Correctness	−0.21	0.05	1.55	B	1.45	A	0.32	0.60
Originality	0.23	0.05	0.78	A	0.76	A	0.45	1.04
Themes	0.08	0.05	0.94	A	0.79	A	0.45	1.12

### Functioning of the items across chess expertise levels

To determine the functioning of the items across participants of different levels of expertise, we explored the Wright Map (Figure 4). The logit scores of the Expert-level chess players were the highest on the SACC. Expert-level chess players were followed by the Advanced players’ category. Beginner-level and Unrated players obtained lower scores than the Advanced category, but higher than Intermediate I and Intermediate II chess rating categories. The Infit and Outfit statistics for chess rating categories were in a range from 0.73 to 1.27 and from 0.45 to 1.03, respectively, which can be interpreted as productive for the measurement (Table 11).

**Table 11:** Fit indices for the chess rating categories.

Category	Measure	S.E.	Infit MSE	Fit category	Outfit MSE	Fit category	Pt Bis	Disc
Unrated	−0.23	0.03	1.00	A	1.00	A	0.35	0.97
Beginners	−0.04	0.07	1.10	A	0.98	A	0.32	0.92
Intermediate I	−0.38	0.06	0.96	A	1.03	A	0.30	0.88
Intermediate II	−0.38	0.06	1.02	A	1.03	A	0.32	0.88
Advanced	0.19	0.05	1.27	A	1.02	A	0.35	1.06
Expert	0.84	0.10	0.73	A	0.45	B	0.19	1.11

## Discussion

The present study aimed to construct and examine the psychometric qualities of the SACC utilizing the Many-facet Rasch model. The content validity of the instrument was supported by mapping chess principles on constructs in creativity and aesthetics literature. The study forwards the following key findings: (a) The Rasch analysis demonstrated good model-data fit and high reliability of the scale; (b) Three expert raters had a high level of agreement across the five dimensions, with acceptable agreement on the dimension of Originality; and (c) There was a significant effect of chess skill level on the functioning of the items.

### Psychometric quality of the SACC

The construction of the SACC was based on the chess, creativity, and aesthetics literature (Amabile, 1983; Cropley and Cropley, 2008, 2011; Damsky, 2002; Iqbal and Yaacob, 2008; Lasker, 1960; Margulies, 1977; Osborn, 1953). Creativity in chess can be defined as what players perceive to be original, surprising, and effective. Although researchers attempted to construct objective measures of chess creativity that would not rely on human raters, subjective components cannot be fully excluded (Iqbal and Yaacob, 2008; Le Lionnais, 1951; Margulies, 1977; Wilson, 1959).

The Many-Facet Rasch analysis demonstrated that the scale was unidimensional and explained 50.59% of the variance in the data, with indices of reliability for items and participants 0.83 and 0.93, respectively. The Wright Map (Figure 4) showed that the Originality item was the hardest to endorse, while Correctness and Wins Economically were the easiest. Creativity studies suggest that both originality and effectiveness are major components of creativity; however, original ideas are rarer. Moreover, when judging the creativity of the products and solutions, human raters tend to have the largest rates of disagreement on the originality component (Acar et al., 2017; Runco and Jaeger, 2012).

### The scale demonstrated a high level of inter-rater agreement

Creativity researchers recommend recruiting raters who have a high level of expertise in the domain (Amabile, 1982). In the present study, we recruited three expert-level chess players to judge the creativity of chess solutions across six chess creativity dimensions (See Table 1). Inter-rater reliability analysis found that the Originality dimension had the lowest level of inter-reliability (*α* = 0.627); however, a similar dimension, Heuristics Violation, had a higher level of agreement among raters (*α* = 0.741).

Originality can be judged on the continuum from Mini-c to Big-C. Mini-c creativity can be defined as intrapersonal creativity in the process of learning (Beghetto and Kaufman, 2007). When one encounters a new problem for which there is no obvious solution, they need to find novel approaches to tackle it. Mini-c creativity might be hard for experts to judge since they need to take into consideration the subject’s skill, experience, and context. As the results of our study demonstrated, in the context of mini-c creativity, violation of the heuristics component is easier to agree on for the experts in comparison to the Originality dimension.

The Correctness component was the easiest for the raters to agree on (*α* = 0.897). Creativity studies have demonstrated that ratings of originality and effectiveness are often negatively correlated (Diedrich et al., 2015; Runco and Charles, 1993). However, the importance of originality and usefulness has been found to depend on the task (Acar et al., 2017; Caroff and Besançon, 2008; Han et al., 2021). In the context of unreal task situations, originality is usually rated higher than in real-life tasks (Runco et al., 2005; Lloyd-Cox et al., 2022). While in the context of science, originality and usefulness have been shown to have equal importance (Long, 2014). Therefore, in intellectual domains such as chess and science, ratings of originality and usefulness have equal importance. Although the human raters are often recruited to judge the creativity of the ideas and products, there might be considerable differences depending on raters’ level of expertise (Barbot et al., 2019). Experts might be more effective in judging the usefulness of the ideas than non-expert raters; however, to rate originality, raters should be aware of the nature of the task and the skill level of the subjects.

### The effect of level of chess expertise on the functioning of the items

The Wright Map has demonstrated that Expert level chess players had the highest scores on the scale, followed by Advanced level players. In line with previous studies on creativity in the field of sports, expert-level players had the highest scores on the SACC (Memmert, 2015; Richard et al., 2017). There is an ongoing debate in the creativity literature regarding the effect of knowledge on creativity (Amabile, 1982; Baer and McKool, 2014), with some studies showing that creativity requires a certain level of expertise (Baer, 2015; Runco, 2004). In the present study, chess skill level significantly correlated with all dimensions of the scale except Violation of Heuristics. Moreover, as the results of Fisher’s z-test demonstrated, the chess rating category had a statistically higher correlation with Correctness than with the Heuristics violation dimension. This finding might imply that expertise plays a more important role in finding effective rather than surprising solutions. As previous studies of chess expertise demonstrated, hours of practice explain only 26% of the variance in chess skill (Burgoyne et al., 2019), while cognitive and personality factors might account for the unexplained variance (Macnamara et al., 2014).

In the present study, Unrated and Beginner level chess players obtained higher scores across chess problems of varying difficulty than the Intermediate category. In line with previous chess studies of the Einstellung effect, intermediate-level players might demonstrate decreased flexibility due to the tendency to give preference to familiar optimal solutions rather than looking for original ones (Bilalić et al., 2008; Sheridan and Reingold, 2017). However, the outperformance of intermediate players by beginners on creativity scores deserves further analysis. There might be an inverted U-shaped relationship between skill level and creativity (Weisberg, 1999). Beginners may produce seemingly creative moves due to a lack of structured knowledge, while intermediate players may become more constrained by learned strategies, before advanced and expert players are able to reintroduce creative flexibility at a higher level of expertise.

### Limitations and future directions

The findings of the study, although provide some valuable insights, should be considered in light of several limitations related to the sample size, raters, and measurement issues. First, the sample was predominantly male, which reflects the historical underrepresentation of women in chess (Chabris and Glickman, 2006). Second, the subsamples of rated chess players are relatively small for making conclusions regarding the relationship between chess creativity and expertise. It also should be noted that the category of unrated chess players is not homogenous in their skill level, since years of practice in the subsample can widely vary. Additionally, in future studies, more expert and non-expert raters should be recruited to examine variability in raters’ judgments depending on the skill level.

The review of literature on chess creativity and aesthetics has demonstrated that the process of creative problem-solving in chess compositions and real games significantly differs (Humble, 1993; Le Lionnais, 1951; Margulies, 1977). In the case of chess composition, players are presented with the end goals, consequently, problems are not entirely ill-defined. While in real chess games, there are numerous possible move sequences to continue the game, and players are not always motivated to find creative solutions. Future studies might explore the differences in creative problem-solving depending on the task structure. Chess problems significantly vary in terms of imposed cognitive load, number of chunks to be operated, and well-defined or ill-defined goals, and the depth of needed search. In addition, creativity and aesthetics studies suggest expanding measurement dimensions by including the elements of feasibility and parsimony in future studies (Friedlander, 2024).

Moreover, in real games, creative combinations are rare and often occur as the result of weak play by the opponent. The awareness regarding the opponent’s skill level and chess personality profile (Dhou, 2018) may also affect how one adjusts strategy in challenging the opponent with original and unexpected chess moves (Leach, 2022). In future studies, we recommend including the variable of hypothetical opponents’ skill levels to examine changes in creative problem-solving depending on the context.

## Conclusion

Measurements of creativity are often criticized for being domain-general rather than domain-specific and being weak predictors of real-life creative achievements (Maker et al., 2023). In the present study, we aimed to construct and validate the SACC. The scale has demonstrated good psychometric qualities with high inter-rater agreement. The finding revealed that the level of expertise in chess is related to chess creativity; however, while Expert and Advanced level chess players had the highest creativity scores, Intermediate level players scored lower than other chess rating categories.

## Data Availability

The raw data supporting the conclusions of this article will be made available by the authors without undue reservation.
